# The Absorption Characteristics of Nonvolatile Components in a Water Extraction From *Amomi fructus* as Determined by *In Situ* Single-Pass Intestinal Perfusion and High-Performance Liquid Chromatography

**DOI:** 10.3389/fphar.2020.00711

**Published:** 2020-06-05

**Authors:** Yuebao Yao, Wenjuan Mi, Guangzhao Cao, Ruiqi Yang, Huirong Chen, Yong Liu, Huiqin Zou, Yonghong Yan

**Affiliations:** ^1^School of Chinese Materia Medica, Beijing University of Chinese Medicine, Beijing, China; ^2^Institute of Chinese Medicine Research, Yangtze River Pharmaceutical Group Jiangsu Longfeng Tang Traditional Chinese Medicine Co., Ltd., Taizhou, China; ^3^Chinese Medicine Research Center, Beijing Increase Innovative Medicine Co., Ltd, Beijing, China; ^4^Department of Clinical Study, Beijing Highthink Pharmaceutical Technology Service Co., Ltd, Beijing, China

**Keywords:** *Amomi fructus*, gastrointestinal diseases, water extraction, *in situ* single-pass intestinal perfusion, high-performance liquid chromatography

## Abstract

**Background:**

*Amomi fructus* is a famous traditional Chinese medicine (TCM) that can exert beneficial effects during the treatment of gastrointestinal diseases and is used widely in China and other countries in Southeast Asia. However, the nonvolatile active ingredients that are present in the water extractions from *A. fructus* used to treat gastrointestinal diseases have yet to be elucidated. The goal of this study was to identify the nonvolatile active ingredients of *A. fructus*.

**Methods:**

We used an *in situ* single-pass intestinal perfusion (SPIP) model to identify the active ingredients of *A. fructus* that play significant roles in gastrointestinal absorption. In addition, we developed a high-performance liquid chromatography (HPLC) method to identify key fractions in intestinal outflow perfusate.

**Results:**

Nineteen components were identified in a water extraction from *A. fructus*; these exhibited different absorption capabilities in different intestinal segments. Of these, six components were determined by the newly developed HPLC method: catechin, vanillic acid, epicatechin, polydatin, isoquercitrin, and quercitrin.

**Conclusions:**

The current study aimed to identify the active ingredients present in water extractions prepared from *A. fructus* in a single-intestinal perfusate from rats. Our findings provide an experimental basis to explain the pharmacodynamic actions of *A. fructus*.

## Introduction

Globally, many patients are required to undergo surgery for benign or malignant gastrointestinal lesions every year. The mortality and complication rates associated with patients undergoing gastrointestinal surgery have decreased over recent years, mostly due to the development of modern medicine and surgical technology. However, gastrointestinal surgery always results in disorders associated with gastrointestinal motility. These disorders vary in severity and are known to be influenced by a wide range of factors, including anesthesia, surgical emergency, trauma, inflammation, and pain, but particularly by radical gastrectomy and other highly traumatic surgeries (Nusrat and Bielefeldt, 2013; Merkow et al., 2015; Reddy et al., 2018).

Conventional forms of western medicine are commonly used to facilitate the treatment of gastrointestinal disease, including fasting, gastric decompression, nutritional support, gastro-kinetic agents (e.g., domperidone, cisapride, and duphalac), and anal preparations for promoting defecation (e.g., glycerin and mannitol preparations). However, while these methods may alleviate clinical symptoms to some extent, they are also associated with adverse drug reactions and high costs.

In recent years, practitioners and researchers of traditional Chinese medicine (TCM) and integrated traditional Chinese medicine (ITCM) have made many valuable clinical observations and carried out numerous experimental studies with regards to the recovery of gastrointestinal function after surgery. It is clearly evident from the existing literature that compound prescriptions of TCM that contain *Amomi fructus* as the predominant medicine are widely used in the treatment of gastrointestinal diseases (Lee et al., 2016). Around 60 different Chinese formulas containing *A. fructus* as the major component are officially documented in *Chinese Pharmacopeia* (2015 version) (Commission of Chinese Pharmacopoeia, 2015).

Previous studies have reported that the water extraction of *A. fructus* can significantly enhance the amplitude of the basic electrical rhythm on electrogastrograms and can also promote intestinal peristalsis. Furthermore, there are clear differences in the resumption of gurgling sounds, along with exhaust and defecation times, when compared between patients receiving water extraction of *A. fructus* and patients receiving conventional forms of modern medicine, such as enteral nutrition solution *via* nasal feeding (Huang et al., 2007; Chen et al., 2018; Suo et al., 2018).

However, previous studies relating to the active components of *A. fructus* are limited to descriptions of volatile oils and other volatile components, such as bornyl acetate, camphor, and limonene (Xu et al., 2018; Ao et al., 2019). In order to fully understand the specific intervental effects of *A. fructus* on the gastrointestinal tract and explore the active ingredients more comprehensively, we cannot ignore other nonvolatile components in the water extraction of *A. fructus*, which may also be important, such as Zn, Mn, other trace elements, vanillic acid, quercetin, piceid, and other water-soluble components (Xue et al., 2016).

*In situ* intestinal perfusion has been used widely to study the absorption of drugs in the intestine (Yerasi et al., 2015; Li et al., 2016; Li et al., 2018; Zhang et al., 2019; Wang et al., 2020). The basic principle of this method is the hypothesis that the reduction of drugs is equivalent to the absorption of drugs and to evaluate the absorption of drugs in the small intestine by reducing drug components in the perfusion solution. Using this method, it is possible to express the level of absorption by the rate of absorption and the amount of drugs absorbed. Moreover, a number of kinetic parameters can be calculated to reflect the absorption of drugs, thus allowing us to analyze the metabolic profiles underlying the permeation and absorption of drugs, such as the effective permeability coefficient (P_eff_) and the absorption rate constant (KA). In this study, we used SPIP as it is an established and effective model for *in situ* drug absorption studies. This model is characterized by a low flow rate (0.2–0.3 ml/min), a stable absorption rate, and causes minimal damage to the mucosa of the intestinal wall (Huo et al., 2014; Lin et al., 2015).

Collectively, the available evidence indicates that conventional western medicine is not fully effective for the treatment of gastrointestinal motility disorders following gastric surgery and hypothesized that TCM and ITCM may provide good therapeutic effects for such patients, particularly considering the widespread application of compound prescriptions of TCM containing *A. fructus* as the main medicine. However, it is highly evident that existing studies relating to the active components of *A. fructus* are limited to descriptions of volatile components; nonvolatile components have yet to be investigated, even though these components may exhibit beneficial pharmacological activity. In the present study, we investigated promising nonvolatile components in water extractions prepared from *A. fructus* in the intestine of rats using the SPIP model and an efficient quantitation method based on high-performance liquid chromatography (HPLC).

## Materials and Methods

### Plant Materials

**Figure 1:**
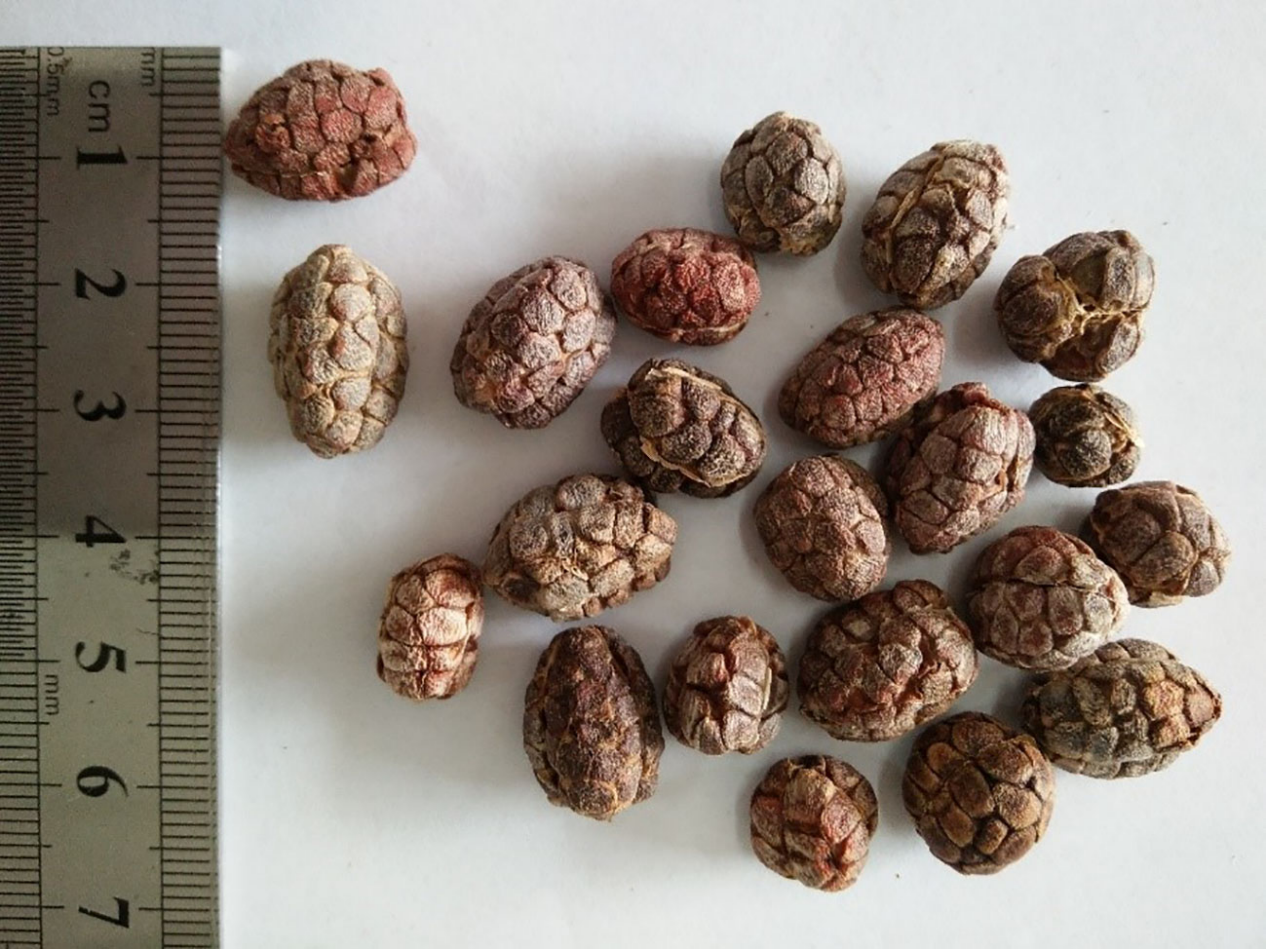
Samples of *Amomi fructus*.

Samples of *A. fructus* (**Figure 1**) were purchased from Anguo Traditional Chinese Medicine Factory (Anguo, China), produced in Guangdong Province. Samples were acquired from the dried ripe fruit of *Amomum villosum* Lour. by Professor Yonghong Yan (Beijing University of Chinese Medicine, Beijing, China). Voucher specimens were then deposited in our laboratory.

### Chemicals and Reagents

We purchased standards for quercetin (purity=98.0%) and vanillic acid (purity=98.0%) from Shanghai Shidande Standard Technical Service Co., Ltd. (Shanghai, China). HPLC-grade methanol (purity≥99.9%) was purchased from Fisher Chemical (Waltham, USA). All water used in this research was double distilled. Other reagents and chemicals were all analytical grade. MgCl_2_·6H_2_O (purity≥98.0%), KCl (purity≥99.5%), NaCl (purity≥99.5%), CaCl_2_ (purity≥96.0%), D-Glucose ([α]20D: +52.5° - +53.0°), NaHCO_3_ (purity≥99.5%), NaH_2_PO_4_ (purity≥99.0%), concentrated hydrochloric acid (ω%36.0–38.0%), and acetic acid (purity≥99.5%) were supplied by Beijing Chemical Works (Beijing, China). Ethyl carbamate (trade name: urethane) was purchased from Tianjin Guangfu Fine Chemical Research Institute (Tianjin, China). Finally, 0.9% sodium chloride injection solution was obtained from Shijiazhuang Pharmaceutical Siyao Co., Ltd. (Shijiazhuang, China).

### Experimental Animals

Male SD rats (clean grade, with a body weight of approximately 250 g) were provided by Sbeifu Biotechnology Co., Ltd. (Beijing, China; License number: SCXK-20160002). The condition of all rats was examined by the Experimental Animal Center of the Academy of Military Medical Sciences (Beijing, China).

### Preparation of Krebs-Ringer’s (K-R) Nutrient Solution

Krebs-Ringer’s (K-R) nutrient solution was prepared by fully dissolving 0.084 g MgCl_2_·6H_2_O, 0.35 g KCl, 7.80 g NaCl, 0.37 g CaCl_2_, 1.48 g Dextrose, 1.37 g NaHCO_3_, and 0.02 g NaH_2_PO_4_ in 1,000 ml double distilled water. The pH of the solution was then adjusted to 7.0 using 1 mol·L^-1^ HCL. The CaCl_2_ was dissolved separately prior to addition to the nutrient solution. This solution was prepared according to a previously described method with some modifications (Wang et al., 2017).

### Preparation of Solutions Containing *A. fructus* for Perfusion

*A. Fructus* was homogenized for 4 seconds using a pulverizer. Traditionally, *A. fructus* is first crushed with or without other medicines. Then, 150 ml of double distilled water was measured into a flat-bottomed 500 ml flask and heated in an induction cooker (power: 2,200 W). Then, the finely homogenized samples (10 g) were weighed and added into the flat-bottomed flask while the water boiled continuously. The heat was then reduced to 400 W for 2 min with the solution continuing to boil gently. Next, the water extraction of *A. fructus* was filtered with a double-layer gauze and the filtrate was centrifuged at 3,000 rpm/s for 20 min to remove *A. fructus* residue. The water extraction of *A. fructus* was then concentrated to 0.5 g/ml by rotary evaporation.

### *In Situ* Single-Pass Intestinal Perfusion

In order to reduce the adsorption of drugs by tubes during the experiment, the tubing of the peristaltic pump was saturated with the perfusion solution until the concentration of the outflow solution was equal to that of the perfusion solution. The rats were fasted for 24 h, during which they were allowed to drink water *ad libitum*. Next, the rats were given intraperitoneal injections of 25% urethane at a dose of 0.007 ml/g. The rats were placed in a supine position on the operating table. Their abdominal cavity were opened along the midline of the abdomen, and the duodenum, jejunum, and ileum were separated from the intestinal segments. Tubes were carefully inserted at either end of this segment and ligated with a sterile surgical line. The intestinal contents were then washed with K-R solution which had been preheated to 37°C. The intestines were then balanced with K-R solution for 15 min and air was pumped into the intestines to drain the residual K-R solution. The intestinal segments were then perfused with the water extraction of *A. fructus* from the peristaltic pump inlet. The small bottles containing the perfusion solution had been accurately weighed in advance. The volume flow was set as 0.2 ml/min, and the absorption was maintained for approximately 30 min. The outflow perfusate was collected with another small bottle which had also been weighed in advance. The bottles containing the perfusion solution and the outflow perfusate bottles were changed quickly every 15 min and weighed. Sampling and weighing were carried out at 45 and 180 min, thus allowing us to calculate the mass of the solution perfused and collected. In situ single-pass intestinal perfusion was implemented in accordance with the literatures with some modifications (Li et al., 2012; Zhou et al., 2012; Jang et al., 2013; Wang and Li, 2018; Xu et al., 2019). At the end of the experiment, the corresponding intestinal segments (duodenum, jejunum, and ileum) were dissected and their length (L) and perimeter (s) were measured. Then, 1 ml of outflow perfusate was carefully transferred and concentrated until they were dry by rotary evaporation. Finally, the concentrate was dissolved in 80% methanol solution and the resulting sample solution was filtered through a 0.22-µm filter before being analyzed by HPLC.

### Conditions for HPLC Analysis

All HPLC analyses were performed on a Waters 2695 HPLC system (Waters Corporation, Milford, MA, USA). Chromatographic separation was carried out with a Waters Sunfire C18 column (5 µm, 4.6 × 250 mm). Elution was carried out by a gradient with a mobile phase consisting of methanol (C) and 0.05% formic acid in water (D) at a flow rate of 1 ml/min. The gradient elution was conducted as follows: 5% C (0.0–10.0 min), 12% C (10.0–24.0 min), 19% C (24.0–37.0 min), 36.6% C (37.0–47.0 min), 40% C (47.0–67.0 min), 36.6% C (67.0–70.0 min), and 70% C (37.0–47.0 min). The column temperature was maintained at 35°C throughout the entire process. The sample size for injection into the HPLC system was set at 20 µl. The detection wavelength was set to 298 nm.

### Method Validation

#### Preparation of Analytical Standard Solutions

Analytical standards for quercetin (1.78 mg) and vanillic acid (3.04 mg) were finely weighed into separate volumetric flasks. These were then adjusted to 10 ml by the addition of 80% methanol. The resultant solutions were shaken up-and-down until well mixed.

#### Preparation of a Standard Curve for Mixed Quercetin and Vanillic Acid Standards

A stock solution of mixed standards was prepared by precisely aliquoting 3 ml of each of the quercetin and vanillic acid standard into an 10 ml Ep tube and shaking up-and-down until the solution was mixed well. A range of other standards were prepared by diluting this stock solution in the following proportions: 1, ½, ¼,1/8, 1/16, and 1/32. All solutions (20 µl in volume) were then filtered through 0.22-µm filters and analyzed by HPLC.

#### Validation of Precision

A stock solution of mixed standards of vanillic acid (600 μl) and quercetin (600 μl) was injected 6 times continuously according to the chromatographic conditions under “2.7”. The peak areas of vanillic acid and quercetin were recorded and the relative standard deviation (RSD) values of vanillic acid and quercetin were calculated.

#### Validation of Method Specificity

Krebs-Ringer’s (K-R) nutrient solution was mixed with the medication solution and allowed to pass through the duodenum, jejunum, and ileum of experimental rats. A blank K-R nutrient solution was also passed through these anatomical structures. Following passage through the intestines of the experimental animals, the test and blank solutions were treated as described in *In Situ Single-Pass Intestinal Perfusion*. These samples were then analyzed by HPLC as described in *Conditions for HPLC Analysis*.

### Data Analysis and Statistics

All data are presented as a mean ± standard deviation (SD). The effective permeability coefficient (P_eff_), as determined by gravimetry, was calculated using Equation (1) in which Vin and Vout are the intestinal perfusate input and output volumes (ml); these were calculated by gravimetric methods, assuming that the density of the perfusion solution at the inlet and outlet was the same. Q represents the perfusion rate (0.2 ml/min), Cin and Cout represent the mass concentrations of the enteric importer and exporter perfusate (μM/L), and 2πrL is the area of the mass transfer surface (cm^2^).

$$\mathrm{Peff}=\frac{-\mathrm{Qln}\left(\frac{\mathrm{Cout}\;\mathrm{Vout}}{\mathrm{Cin}\;\mathrm{Vin}}\right)}{2\pi \mathrm{rL}}$$

## Results

### Chromatographic Study

#### The Standard Curve for the Quercetin and Vanillic Acid Mixed Standards

Standard curves for quercetin and vanillic acid were performed for peak area (Y) and the quantity of standard solution injected (X), respectively:

Y=20696360.51X−6341.07 (r =1.0000) (quercetin)

Y=31305076.57X−39681.19 (r=1.0000) (vanilic acid)

The linearities for quercetin and vanillic acid in the working standard solutions (0.0028–0.0890 mg/ml, 0.0048–0.1520 mg/ml) was good, as demonstrated by the fact that their correlation coefficients (r) exceeded 0.9999.

#### Precision

The RSD values for peak area response were 0.31% (vanillic acid) and 0.17% (quercetin). These figures indicated that the HPLC method showed very good levels of precision for our analysis under the specified conditions.

#### Specificity

**Figure 2:**
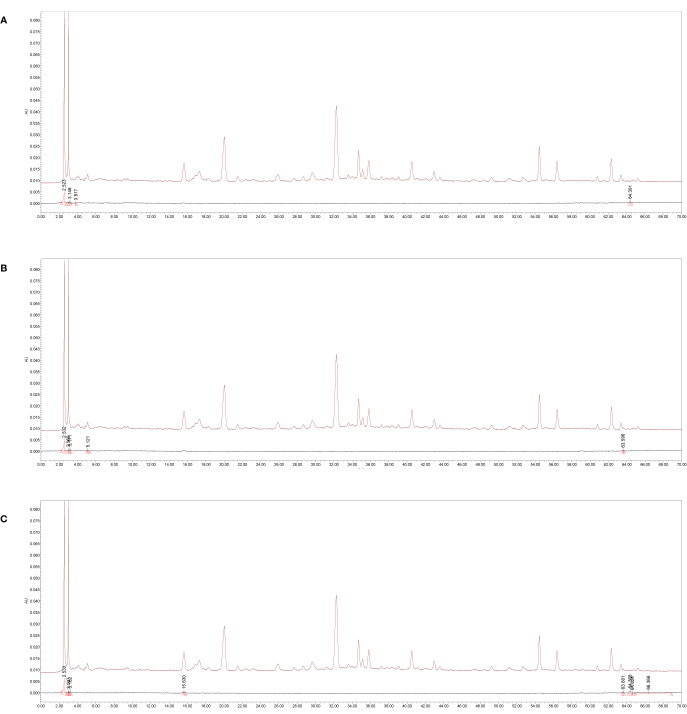
The specificity high-performance liquid chromatography (HPLC) chromatograms of absorption samples of water extraction from *Amomi fructus* in single-pass intestinal perfusion (SPIP). **(A)** Blank Krebs-Ringer’s (K-R) nutrient solution and K-R nutrient solution mixed with medicine solution *via* duodenum, **(B)** blank K-R nutrient solution and K-R nutrient solution mixed with medicine solution *via* jejunum, **(C)** blank K-R nutrient solution and K-R nutrient solution mixed with medicine solution *via* ileum.

Chromatograms showed that the blank K-R nutrient solution that had passed through the duodenum, jejunum, and ileum did not interfere with the chromatographic peaks of the tested samples (**Figure 2**).

#### Determination of the Characteristic Components of *A. fructus*

**Figure 3:**
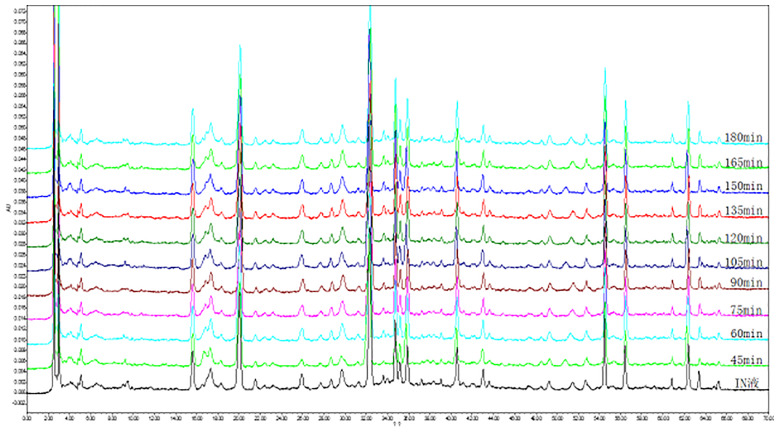
High-performance liquid chromatography (HPLC) chromatogram of the water extraction of *A. fructus* at different time *via* jejunum.

**Figure 4:**
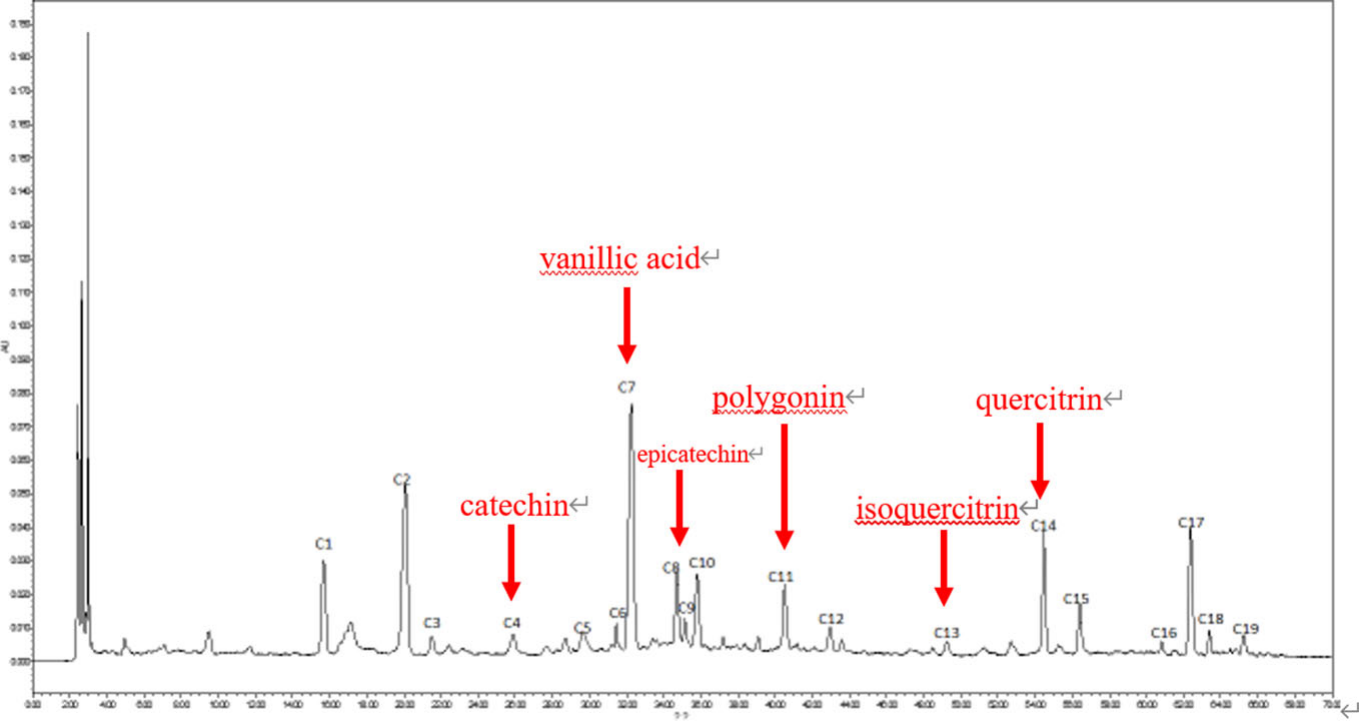
High-performance liquid chromatography (HPLC) chromatograms of analytical standards.

We wanted to investigate the metabolic conditions of the K-R solution containing water extraction from *A. fructus* in different intestinal segments within 3 h of passage. In order to do this, we first needed to produce HPLC chromatograms of the water extraction of *A. fructus*. As shown in **Figure 3**, we observed 19 characteristic peaks in the HPLC chromatograms produced by *A. fructus* water extractions. A total of six compounds (C4, C7, C8, C11, C13, and C14) were identified on the HPLC chromatograms by the use of analytical standard solutions (**Figure 4**): these compounds were identified as catechin, vanillic acid, epicatechin, polygonin, isoquercitrin, and quercitrin, respectively.

### *In Situ* Single-Pass Intestinal Perfusion

First, we used standard curves to calculate the concentrations of quercetin and vanillic acid. The contents of the other unknown components were calculated by using the concentration of vanillic acid as a reference. In this study, we used the weight analysis method to correct the inflow and outflow volume of the perfusion solution and eliminate the influence of volume change; we then calculated the P_eff_ of each component over 180 min (**Table 1**).

**Table 1:** P_eff_ of 19 components in different intestinal segments of rats (x ± s, n = 3).

NO.	P_eff_/×10^-4^cm·s^-1^		
	Duodenum	Jejunum	Ileum
C1	0.7281 ± 1.360	0.4401 ± 1.487	0.4457 ± 3.051
C2	0.2233 ± 0.5947	0.2035 ± 1.665	0.2485 ± 2.095
C3	2.934 ± 3.972	1.971 ± 4.479	1.850 ± 2.858
C4	0.2551 ± 5.158	0.1572 ± 1.586	0.4204 ± 4.496
C5	2.008 ± 6.887	1.970 ± 9.507	0.9870 ± 6.072
C6	1.278 ± 13.29	2.115 ± 10.31	1.377 ± 10.70
C7	0.6886 ± 4.326	0.5706 ± 0.9930	0.4668 ± 5.272
C8	0.7866 ± 6.146	0.4894 ± 4.184	0.5355 ± 5.257
C9	0.005700 ± 3.707	-0.03380 ± 4.586	0.3553 ± 3.583
C10	0.04790 ± 3.124	-0.01680 ± 1.307	0.08590 ± 4.283
C11	0.4034 ± 4.664	0.3610 ± 1.338	0.3361 ± 5.402
C12	3.442 ± 11.40	4.156 ± 12.62	0.6705 ± 9.997
C13	0.5995 ± 1.471	0.5268 ± 2.324	0.9981 ± 2.987
C14	0.2754 ± 0.7495	0.1101 ± 1.658	0.4668 ± 2.609
C15	0.00040000 ± 7.981	-0.07570 ± 4.696	0.4491 ± 8.277
C16	0.5032 ± 9.868	0.2538 ± 4.239	0.9350 ± 9.101
C17	1.426 ± 5.311	1.127 ± 2.961	1.033 ± 5.946
C18	0.2643 ± 25.52	-0.1435 ± 10.23	0.9447 ± 21.72
C19	0.5109 ± 14.59	-0.06070 ± 10.18	1.226 ± 12.63

Red font = strong absorption; blue font = moderate absorption; green font = poor absorption; black font = negative value.

**Figure 5:**
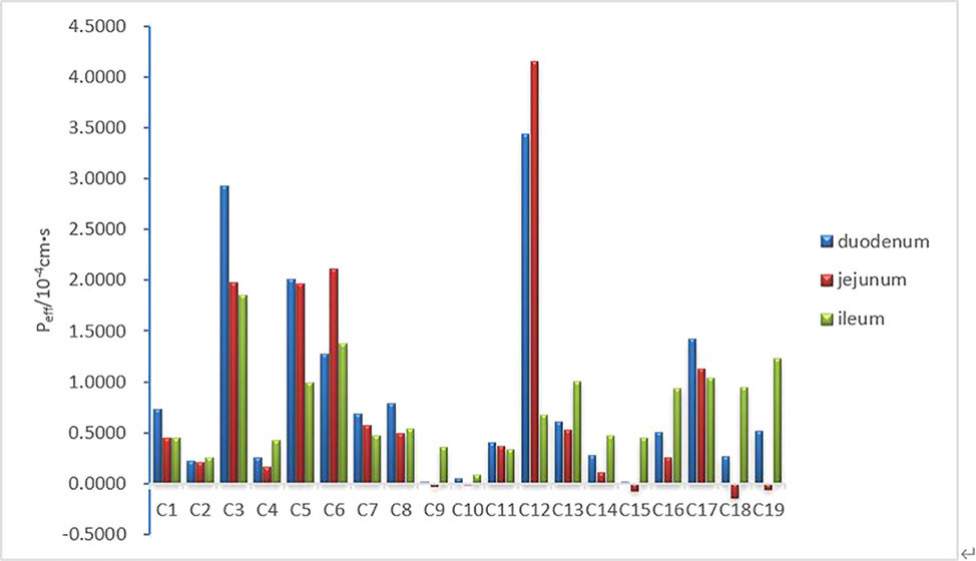
Compare the P_eff_ of 19 components obtained from single-pass intestinal perfusion (SPIP) perfusion.

The principle of single-pass intestinal perfusion is that the measured reduction in drug concentration is equal to amount that has been absorbed. Generally, absorption performance can be classified into three groups by calculating P_eff_. When P_eff_ < 3×10^-6^ cm/s the drug absorption is said to be poor, while a P_eff_ > 2×10^-5^ cm/s indicates good drug absorption; between these two values, drug absorption is said to be moderate (Fagerholm et al., 1996). Based on these criteria, we found that the 19 components in *A. fructus* water extraction showed different absorption capabilities in different segments of the intestine (**Figure 5**). Components Cl, C2, C3, C4, C5, C6, C7, C8, C11, C12, C13, C14, C16, C17, C18, and C19 showed strong absorption in the duodenum. Component C10 showed moderate absorption in the duodenum, while components C9 and C15 showed poor absorption in the duodenum. The absorption characteristics of the 19 components in the jejunum are shown in **Table 1**; components Cl, C2, C3, C5, C6, C7, C8, C11, C12, C13, C16, and C17 showed strong absorption, while C4 and C14 showed moderate absorption. It is worth highlighting that negative P_eff_ values were obtained for C9, C10, C15, C18, and C19, suggesting that these five components were enriched in the jejunum. We also discovered that all of the 19 components could be absorbed in the ileum, except for the C10 component which showed moderate absorption. Other components showed absorption in the ileum. Generally, most components were absorbed in the ileum; this was followed by the duodenum and then the jejunum. The absorption capacity of the three intestinal segments to the C3, C5, C7, C11, and C17 components was highest in the duodenum, followed by the jejunum and then the ileum. The absorption capacity of the C2, C4, C10, C13, C14, C16, C18, and C19 components was highest in ileum, followed by the duodenum and then the jejunum. The absorption capacity of the C1 and C8 components was highest in the duodenum, followed by the ileum and the jejunum while the absorption capacity of the C6 component was strongest in the jejunum, followed by the ileum and then the duodenum. Finally, the absorption capacity for component C12 was highest in the jejunum, followed by the duodenum and the ileum. Components C9 and C15 were absorbed only in the duodenum; absorption as strong in in this segment for these components.

## Discussion

*A. fructus* is used as a common medicine in TCM and also as a supplement, dietary agent, food additive, and spice in China and other Southeast Asian countries, such as Thailand, Vietnam, Burma, and Indonesia. *A. fructus* is generally boiled in water; the decoction in TCM arose from the Tang Dynasty over 1,300 years ago. This highlights the fact that *A. fructus* contains nonvolatile components that can be dissolved in water and absorbed through the gastrointestinal tract in order to exert their medicinal effect. A number of studies have proven that the water extraction from *A. fructus* exerts beneficial effects in the treatment of inflammation, cancer, and is also able to maintain the balance of the intestinal microecology (Nagare et al., 2010; Choi et al., 2015; Wu et al., 2019). However, previous studies of *A. fructus* only involved descriptions of volatile components, thus restricting its further development as a medicinal agent. Studies on the *in vivo* metabolism of *A. fructus* have not been reported previously. It is also uncertain which of the water-soluble components of *A. fructus* can be absorbed by the intestine. Therefore, this study aimed to explore the absorption characteristics of the nonvolatile components contained within *A. fructus* using *in situ* single-pass perfusion methodology.

Our results revealed there were 19 water-soluble components of *A. fructus* which showed different levels of absorption in different segments of the intestine. interestingly, components C9, C10, C15, C18, and C19 showed negative P_eff_ values in the jejunum. We hypothesize that there were two reasons for this. Firstly, that some large polar molecules in the *A. fructus* water extraction had become degraded, such as glycosides, or that the polymers of these five components had been hydrolyzed to form smaller molecular compounds, thus increasing the contents of these components. Secondly, it is possible that the 5 components with negative values had not been metabolized or absorbed by the jejunum.

Most oral preparations are usually absorbed from the small intestine and exert effect before even reaching the blood stream (Kuang et al., 2017). In the pre-experiment, we investigated the stability of components in the water extraction of *A. fructus* using an artificial gastrointestinal solution. We found that these components were stable in the artificial gastrointestinal solution and did not metabolize or transform; collectively, these findings allowed us to establish the single-pass intestinal perfusion experiment described herein. In addition, our present results showed that quercetin and vanillic acid were present in the water extraction of *A. fructus*. Vanillic acid and quercetin were also shown to exist in the perfusion solution, as verified by a pre-experiment. The perfusion solution featured relatively high concentrations of these two components. Therefore, this study aimed to determine the concentrations of quercetin and vanillic acid using standard curves. The relative concentrations of the other components in the perfusion solution at the different segments were based on the concentration of vanillic acid.

## Conclusion

In the current study, we used an integrated approach based on SPIP and HPLC to identify the absorption characteristics of nonvolatile components in *A. fructus*. We successfully identified six chemical constituents using this approach. Collectively, our data indicate that the use of SPIP in a rat model provided a good estimation of the effective permeability and site of intestinal absorption for the nonvolatile components in *A. fructus*. Our results provide further insight into the therapeutic composition and absorption characteristics of *A. fructu*s and provide a foundation from which to perform research and development to promote its wider clinical application.

## Data Availability Statement

All datasets generated for this study are included in the article/supplementary material.

## Ethics Statement

Animal experiments were conducted in accordance with the guidelines for animal experiments. The animal study was reviewed and approved the Animal Ethics Committee, Beijing University of Chinese Medicine.

## Author Contributions

YuY analyzed the data and prepared the paper. WM and GC carried out the pharmacological experiments. WM arranged all of the experiments. RY conducted physical and chemical analyses. HC processed the samples. YL designed the study and provided theoretical guidance. HZ revised the manuscript and read and approved the final manuscript. YoY administered the project.

## Funding

This study was financially supported by the National Natural Science Foundation of China (Grants nos. 81573542, and 81403054) and Beijing University of Chinese Medicine (Grants nos. 2019-JYB-JS-006).

## Conflict of Interest

WM was employed by company Yangtze River Pharmaceutical Group Jiangsu Longfeng Tang Traditional Chinese Medicine Co., Ltd. RY was employed by company Beijing Increase Innovative Medicine Co., Ltd. HC was employed by company Beijing Highthink Pharmaceutical Technology Service Co., Ltd.

The remaining authors declare that the research was conducted in the absence of any commercial or financial relationships that could be construed as a potential conflict of interest.
